# The Association Between Psychosocial Factors and Decision Making Regarding Primary Treatment in Older Women With Early‐Stage Breast Cancer

**DOI:** 10.1002/pon.70240

**Published:** 2025-07-23

**Authors:** G. Hindler, I. Alabaster, R. Zahit, A. Jahan, D. Giza, H. M. Holmes, H. Blake, K.‐L. Cheung, R. M. Parks

**Affiliations:** ^1^ Nottingham Breast Cancer Research Centre University of Nottingham Nottingham UK; ^2^ Breast Unit King's Mill Hospital Sherwood Forest Hospitals NHS Trust Sutton‐in‐Ashfield UK; ^3^ Division of Geriatric and Palliative Medicine University of Texas Health Science Centre McGovern Medical School Houston Texas USA; ^4^ School of Health Sciences University of Nottingham Nottingham UK

**Keywords:** breast cancer, cancer, cancer treatment, geriatric assessment, oncology, psycho‐oncology, psychosocial support systems, quality of life, surgery

## Abstract

**Background:**

Breast cancer is increasingly prevalent among older adults, who are likely to have numerous comorbidities and unique psychosocial challenges.

**Aims:**

The aim of this study was to measure the prevalence of psychosocial factors in a cohort of older women diagnosed with early‐stage operable breast cancer and the influence these factors may have on treatment decisions.

**Methods:**

As part of a prospective study in three UK centres, 199 patients with a new diagnosis of early‐stage operable primary breast cancer, aged ≥ 70 years (mean 77, range 68–93) were recruited. A cancer‐specific Comprehensive Geriatric Assessment (CGA) was conducted within 6 weeks of diagnosis. Association between treatment decision and psychosocial aspects (as measured by the ‘psychosocial support’, ‘social activity’ and ‘social support’ domains) of the CGA was determined. Treatment decision was not guided by this study and was determined usual conventional methods as per the breast multi‐disciplinary team.

**Results:**

Scores for ‘psychosocial support’ averaged 82.1/102, ‘social activity’ averaged 13.5/24, and ‘social support’ averaged 43.3/72; with a higher score indicating a more positive outcome. There was no association between total scores in these domains and the type of treatment received. A lower score in three individual questions was associated with a higher likelihood of non‐surgical treatment.

**Conclusions:**

While no direct link emerged between overall psychosocial scores and treatment decisions using CGA, specific sub‐questions displayed associations with non‐surgical treatment. This study is the only one of its kind to our knowledge. This may have implications for the design of a pre‐CGA screening tool.

## 1 Background

Breast cancer ranks highest by incidence amongst new cancer cases, accounting for 15% of all new cancer cases [1]. Age is strongly related to the incidence of breast cancer, with rates rising steadily from around age 30–34 and more steeply from around age 70–74 [2]. Discounting breast cancer status, older patients tend to be more frail and have more comorbidities [3]. Older adults with multiple chronic conditions are known to vary in their most desired health outcomes and in the health care that they are willing and able to receive [4]. Therefore, these conditions may affect cancer treatment uptake, tolerance, and clinical outcomes.

Studies have been conducted to investigate the impact of different treatment methods of breast cancer on quality of life (QoL), however the specific psychosocial impacts of these different treatment methods have not been investigated in detail, especially in older women [5, 6] Older patients may exhibit several factors that may add psychological burden to their breast cancer journey, such as decreased social support, clinical bias towards undertreatment and financial burden [3, 7]. As such, the investigation of the psychosocial impacts of various treatment methods, specifically on older patients, can help to refine patient care.

### 1.1 Distinction Between QoL and Psychosocial Wellbeing

Recent trends in healthcare place more emphasis on Quality of Life (QoL) than curative intent, as the number of research articles investigating health related QoL from 2000 to 2019 has been increasing, particularly articles related to cancer [8]. QoL is a multifaceted idea which encompasses physical, psychological and social wellbeing analogous to psychosocial welfare [9]. However, in practice QoL is more narrowly used in healthcare research to measure the impact of a medical condition over time [10].

Individuals with cancer have far higher rates of psychological distress than the general population [11]. Factors contributing to this include limited social support, chronic medical comorbidities and a prior history of poor psychological health [12, 13].

Utilising multidisciplinary processes such as Comprehensive Geriatric Assessment (CGA) may help us to better gauge psychosocial wellbeing within the older adult.

### 1.2 Comprehensive Geriatric Assessment

CGA is a multidimensional process involving clinical assessment, functional and mental status evaluation, consideration of social circumstances and environmental factors. CGA encompasses a comprehensive evaluation that goes beyond clinical and physical aspects, emphasising psychological and social factors. As part of the CGA, an assessment is made regarding an older patient's social environment and circumstances. It considers an individual's emotional state, social support, coping mechanisms and overall mental health [14].

CGA could potentially be useful in guiding treatment decision‐making for older breast cancer patients' helping to choose the best treatment approach based on comorbidities and life‐expectancy, guiding decision‐making and optimizing them prior to intervention. Barriers to implementing CGA in clinical practice include the time required to complete the assessment, associated costs and how to act on the findings [14, 15].

The overarching goal of the present study is to investigate the relationship between psychosocial wellbeing, as measured by using a modified Hospital Anxiety and Depression Scale (HADS), MOS Social Activity Limitations Measure and MOS Social Support Survey and primary treatment decision in older women with early‐stage operable breast cancer, using a validated cancer‐specific CGA tool. The study's objectives are to (1) Evaluate the cohort's overall psychosocial wellbeing and (2) Investigate the association between psychosocial wellbeing score (as measured by CGA) and primary breast cancer treatment received.

## 2 Methods

This study is a part of an ongoing prospective research conducted in older women diagnosed with primary operable breast cancer in three UK centres. The study design has been described in detail previously and is summarised below [16].

### 2.1 Participant Recruitment

Women aged 70 or older who were newly diagnosed with clinically early operable primary breast cancer and met the below criteria were invited to participate. Treatment decisions were made independently of the study and not guided by any aspect of the CGA. Participants had to be at least 70 years old with a new diagnosis of early operable breast cancer. Patients with prior breast cancer treatment, evidence of metastatic disease, or who were unable to provide informed consent were excluded. One patient aged 68 was included due to multiple comorbidities and considered by the Multi‐Disciplinary Team (MDT) to be ‘older’ [17].

Of the patients approached, approximately 80% of patients approached agreed to participate in the study. In addition, patients in this cohort had similar clinical features and were all eligible for either PET or surgery. This cohort included patients of stage 1 or 2 primary breast cancer, who were ER positive. Patients in this cohort were recruited in the East Midlands; thus, some socioeconomic difference may be expected, however this data was not collected.

Within our cohort, we only have approximate figures for the number of patients were approached before primary treatment had commenced. Therefore, this aspect has not been considered for analysis.

Data collected as part of this study was not used to guide treatment decision, which were already made in the conventional manner by the breast multi‐disciplinary team in conjunction with the patient.

#### 2.1.1 CGA Measures Utilised

The study used a cancer specific validated CGA which was developed by Dr A Hurria, who validated this particular CGA in predicting chemotherapy outcomes for geriatric patients [18]. As part of the present study, the CGA was administered within 6 weeks of diagnosis. On completion of the CGA, in addition to demographic and clinical factors including number of medications and comorbidities, key domains that are of particular interest to this present study were extracted. These domains included psychosocial wellbeing and treatment decision. Psychosocial wellbeing was assessed using the Hospital Anxiety and Depression Scale (HADS) to assess psychosocial function, MOS Social Activity Limitations Measure to assess social activity, and MOS Social Support Survey: Emotional/Information and Tangible subscales for social support [19]. The HADs score in particular was chosen for its well‐established reliability and ease of use in identifying oncology patients needing psychiatric evaluation. This is particularly relevant in oncology patients, where anxiety and depression are common co‐morbidities [20].

These elements as taken directly from the HADS are given in Supporting Information S1: Appendix 1. Standard scoring guidelines were used to score each of the psychosocial wellbeing measures. Total scores for each domain were considered as well as scores for individual questions [18]. Treatment decision was defined as surgical (mastectomy or breast‐conserving surgery) or non‐surgical (primary endocrine therapy) or primary radiotherapy. Hormone receptor status or any other clinicopathological data was not included in this data. Generally, as a cohort of older women with breast cancer, the majority will be hormone receptor positive and human epidermal growth factor receptor 2 negative.

Given that the study includes patients who underwent mastectomy and wide local excision, it is important to consider that these procedures may carry different psychological burdens. Previous research has indicated that younger patients who undergo mastectomy tend to have worse psychological scores compared to those who undergo breast conservation therapy. This potential disparity in outcome is acknowledged as a limitation of this study [21].

For the purposes of this study, an adapted HADS was used, including the Beck Depression Inventory, Spielberger State‐Trait Anxiety Inventory, Clinical Anxiety Scale, and Symptom Checklist 90 Scale, which demonstrate medium to strong correlations with HADS [19]. Functional status was evaluated using a self‐reported measure based on the Karnofsky Performance Status (KPS) scale. Patients rated their own capacity from ‘normal activities’ to ‘severely disabled’. This version, validated in oncology populations, is significantly associated with survival (*P* < 0.05) and provides independent insights from physician assessments [19].

### 2.2 Statistical Analysis

The data obtained from the adapted HADS were coded, de‐identified, and analysed using the Statistical Package for the Social Sciences (SPSS). Descriptive data were treated as categorical variables. Significance was set at *p* < 0.05. The association between each of the three psychosocial wellbeing scales and treatment decision was calculated using Fisher's exact test.

## 3 Results

### 3.1 Background

The total number of participants was 199, the median age of which was 77 years old (range 68–93). Of the 199 participants, 69 (35.9%) underwent mastectomy, 93 (48.4%) has breast conserving surgery, 29 (15.1%) had primary endocrine therapy and 1 (0.5%) had primary radiotherapy. Overall, 162 (81.4%) underwent surgical treatment and 30 (15.1%) had a form of non‐surgical treatment (primary radiotherapy or endocrine therapy). A breakdown of the demographics of the cohort is shown above, as is the self‐rated performance by age bracket and average number of daily medications (Table 1).

**TABLE 1:** Background cohort information where average performance is on a self‐reported scale of 0–8, 8 being best performance.

Age at time of diagnosis	68–74	75–80	80–93	Total
Number	68	78	53	199
Percentage	34.2%	39.2%	26.6%	100%
Average no. of daily meds (range)	3.6 (0–15)	4.5 (0–20)	5.1 (0–13)	4.4 (0–20)
Average performance (range)	6.6 (2–8)	6.7 (2–8)	6.2 (2–8)	6.5 (2–8)

### 3.2 Overall Measure of Psychosocial Wellbeing

Three measures within the HADS: ‘Measures of psychosocial functioning’, ‘Measures of social activity’ and ‘Measures of social support’ (Supporting Information S1: Appendix 1), were analysed to determine whether there is any statistically significant correlation between these, and the type of treatment received (Table 2). The results are discussed individually below.

**TABLE 2:** The maximum total score for all three HADS Domains, the average score overall and the average for each treatment group with range.

CGA domain	Max. Total score	Avg. Score (range)	Avg. Score surgical patients (range)	Avg. Score non‐surgical patients (range)	*p* value
Measures of psychosocial functioning	102	82.9 (46–102)	83.5 (46–102)	79.6 (50–101)	0.539
Measures of social activity	24	16.3 (4–20)	16.4 (4–20)	15.9 (5–18)	0.901
Measures of social support	72	55.9 (12–60)	55.7 (12–60)	56.9 (14–60)	0.466

#### 3.2.1 Association Between Psychosocial Wellbeing (as Measured by HADS) and Primary Treatment Received

**FIGURE 1:**
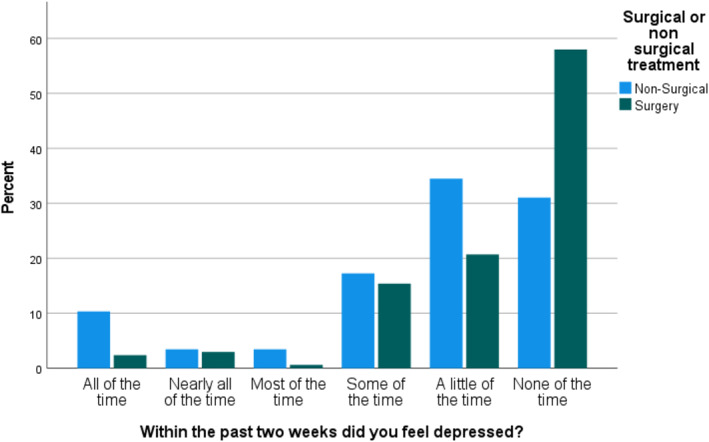
Comparison of responses to ‘Within the past 2 weeks did you feel depressed?’ between surgical and non‐surgical groups.

**FIGURE 2:**
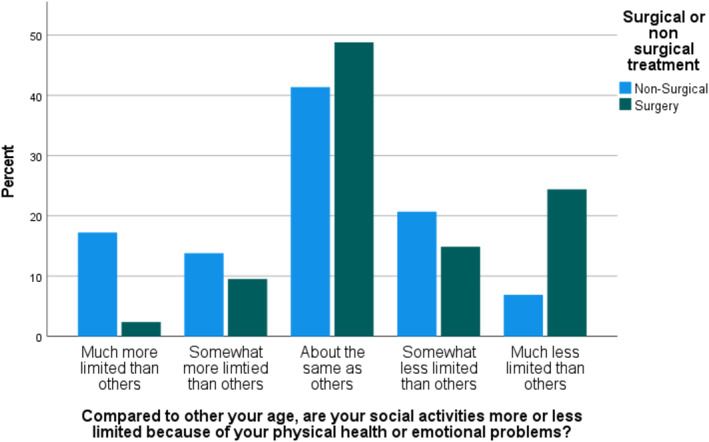
Comparison of responses to ‘Compared to others your age, are your social activities more or less limited because of your physical health or emotional problems?’ between surgical and non‐surgical groups.

**FIGURE 3:**
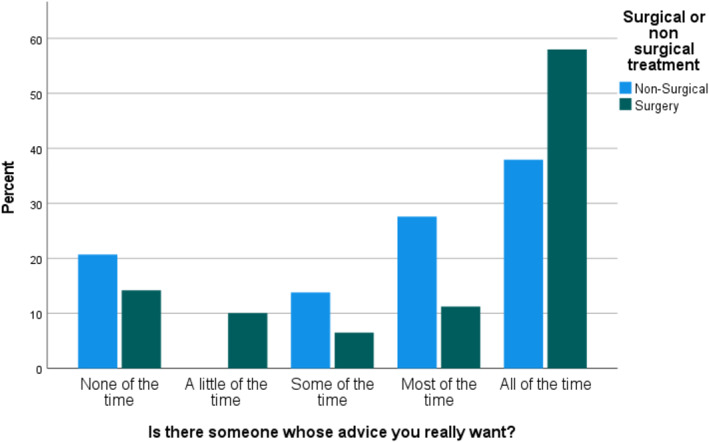
Comparison of responses to ‘Is there someone whose advice you really want?’ between surgical and non‐surgical groups.

No significant association was found between the total score in each of the psychosocial domains and the treatment the patient received. When evaluating each question from each of the three psychosocial wellbeing tools (Supporting Information S1:Appendix 2), most of the individual questions showed no association with treatment decision; however, three questions were found to be correlated with treatment: Within the past 2 weeks did you feel depressed?’ (*p* = 0.019), ‘Compared to other your age, are your social activities more or less limited because of your physical health or emotional problems?’ (*p* = 0.005) and ‘Is there someone whose advice you really want?’ (*p*= 0.012) (Figures 1, 2, 3).

As shown in Figure 1, when answering the question ‘Within the past 2 weeks did you feel depressed?’ (*p* = 0.019) the group of patients in the non‐surgical group felt more depressed ‘all the time’ in the last 2 weeks than the surgical group. The responses to ‘Compared to other your age, are your social activities more or less limited because of your physical health or emotional problems?’ (*p* = 0.005) similarly shows that the non‐surgical group felt more socially limited than the surgical group. Likewise, within the responses to ‘Is there someone whose advice you really want? (*p* = 0.012), more of the surgery group answered ‘all of the time’ than the non‐surgery group.

At the extremes of emotion, that is, ‘all of the time’ and ‘none of the time’, all three questions showed more stark differences between the surgery and non‐surgery groups.

## 4 Discussion

### 4.1 Overall Measure of Psychosocial Wellbeing

Overall, in this prospect cohort of 199 women with early‐stage primary breast cancer undergoing a cancer‐specific questionnaire as part of a comprehensive geriatric assessment (CGA), we found good levels of psychosocial wellbeing. Our assessment of psychosocial wellbeing included three domains: psychosocial functioning, social activity and social support, using validated tools applied in CGA previously. There were high average scores across each of these three domains in our study. Most women participating in this study felt positive, well supported and had enough social interaction to meet their needs. Analysing the averages by treatment group revealed minimal variation in this trend; mental wellbeing was consistently reported as generally good in both groups. Including measures such as these are particularly important as previous studies have shown that social support and activity are strongly associated with lower mortality rates [22, 23, 24].

Previous studies have found that women, specifically older women, experiencing breast cancer treatment have good psychosocial functioning scores. Tesch et al. [25] found that young women with breast cancer report greater psychosocial morbidity and poorer quality of life than older women. Similarly, Naik et al.‘s [26] found that younger women were more likely to report symptoms of depression, anxiety and issues with intimacy, compared to older women with breast cancer.

However, other studies highlight the psychosocial challenges confronting older women. Dinapoli et al.‘s [6] review investigating the psychosocial impact of breast cancer found that older women faced decreased social support, clinical bias towards undertreatment and the need for increased time and care in medical communication. Furthermore, older women may be less disposed to seek psychological or medical help, exhibit higher levels of hopelessness and fatalism and report more concerns about disfigurement and finances [6, 27]. These factors can result in delays or avoidance of treatment. This group experiences a particulary high treatment burden, encompassing health, financial, psychological and social factors [28]; this can affect their priorities for care, leading to compromises in treatment choice.

Given the overall high scores for psychosocial factors in our cohort, these results seem to align with those of Naik et al., and Tesch et al. [25, 26], however we cannot comment on the specific influence of age due to the lack of a younger control group. Reasons behind why the participants in our study scored highly on psychosocial functioning may be due to inherent selection bias in such studies, as discussed in Limitations, or the timing of our questionnaire—within 6 weeks of diagnosis. A post‐treatment assessment, 6 months or a year later, may reflect different scores.

### 4.2 Association Between Psychosocial Wellbeing (as Measured by CGA) and Primary Treatment Received

No association between the overall psychosocial wellbeing scores and treatment decision was noted in this study, as measured by the three subsections of our questionnaire. Only when we evaluated individual constituent items did we see correlations between psychosocial wellbeing and surgical versus. non‐surgical treatment. The question ‘Within the past 2 weeks did you feel depressed?’ is one of the three that showed positive predictive power in our study (*p* = 0.019). This is supported by prior data showing that this question performs well as one half of a simple screening tool for psychological wellbeing in older adults [29].

Prior studies have shown that receiving non‐surgical intervention for breast cancer is associated with greater frailty, age, reduced ability to perform instrumental activities of daily living (IADLs such as planning own meals or using the phone) and activities of daily living (ADLS such as getting dressed or washing oneself) [30, 31]. Indeed, the International Society of Geriatric Oncology (SIOG) and the European Society of Breast Cancer Specialists (EUSOMA) recommend that primary endocrine therapy (PET) should primarily be considered in patients with a 2–3 year life expectancy and who are unfit for or prefer not to undergo surgery [32]. Consequently, our original hypothesis was that lower mood, less social activity and support was likely to be correlated with receiving PET or radiotherapy. Similarly, higher anxiety in the PET group due to worry about the higher likelihood of the return of their cancer could influence the results. However, our study found no clear difference in psychosocial measures between the surgical and non‐surgical groups. This agrees with some of the existing literature.

Husain et al. [33] completed a study focussed on the patient's perspective of their treatment choices. They found that overall, both therapies were well tolerated and had high satisfaction rates from most of their participants. This was true even for women who suffered complications post‐surgery, or if they were in the PET group, changed management due to side effects or other factors. This study also mentioned a strong level of social support found in both groups, unaffected by PET or surgical treatment. Similarly, Jeon et al. [34] found in their 2021 article that there was no correlation between poor psychosocial functioning and choice of surgery. The median age of this cohort was 52.9 however, significantly lower the median age of 77 in the present study.

Nevertheless, other previous literature has found that patients receiving PET rather than surgery have greater QoL outcomes [5]. Indeed, Chia et al. [35] suggest that there is evidence of deterioration in the ability to perform activities of daily living and QoL in older women after breast cancer surgery. This may be because of loss of function in the affected arm after surgery, difficulty recovering or body image issues. Our study shows that greater frailty at the outset may not contribute as much as we thought to lower psychosocial score and it may be that an acute drop in function, such as after surgery, may affect these scores more significantly.

For example, Parks et al.‘s [36] study of 40 breast cancer patients, did find a significant difference between surgical and non‐surgical groups in psychosocial measures. Participants who had non‐surgery rather than surgical treatment had a higher likelihood of better social functioning scores (*p* = 0.032) and better body image scores (*p* = 0.001) after 6 months. Furthermore, non‐surgical patients were more likely to report increased pain (*p* = 0.048) and greater financial difficulties (*p* = 0.001), compared to surgical patients after 6 months.

Wyld et al.‘s [5] study also found a difference between surgical and non‐surgical psychosocial outcomes. In this multi‐centre, prospective observational study, outcome measures (overall and breast cancer specific survival, QoL and adverse events) were recorded at 6 monthly intervals for 2 years. The study included 3416 women from 56 UK breast units between 2013 and 2018. In this study, the frailer participants in the surgical cohort had decreased QoL scores and functional outcomes post‐surgery.

However, it should be noted that Harrison et al.‘s [37] systematic review focussing on the impact of breast surgery on the functional status of older women shows that there is a lack of high‐level evidence on this issue and more randomised controlled trials need to be conducted to generate more high‐quality data to definitively comment on this topic.

The present study supports the conclusions that both surgical and non‐surgical groups had similar overall psychosocial functioning [16, 33, 34], although certain questions in our study did show a statistically significant relationship.

### 4.3 Clinical Implications

This study suggests that psychosocial factors may not be strong predictors of treatment type in older women with breast cancer. Ergo, this section may be limited or reduced in size when designing CGA pre‐screening tools for treatment‐specific geriatric assessment. The three surveyed questions that did show a statistical link to treatment type could be considered for such a tool.

However, there is some evidence for using psychosocial wellbeing tools as part of psychological prehabilitation [38]. Instead of informing treatment decisions, a psychosocial screening tool may be used pre‐treatment as a method of picking up poor psychological health enabling early intervention and thus increasing QoL outcomes.

## 5 Limitations

One of the challenges of CGA is how scores should be interpreted as ‘good’ or ‘bad’ as these are highly subjective. Responses in this analysis were evaluated relative to the overall scores within the cohort. Patients who did not have the capacity to give informed consent because of dementia or other health factors were not included in this study [16], therefore we may have selected a fitter cohort with fewer negative psychosocial factors. Tumour type such as hormone receptor status have not been included as part of this analysis. The cohort is relatively small, and we have conducted several statistical tests. We did not apply a Bonferroni correction in this paper so as not to compromise the power of the study and therefore it may be subject to type 1 error [39]. As previously mentioned we did not have precise figure for how many in our cohort were pre and post treatment therefore this did not factor into our analysis.

In addition, the conflation of breast surgeries in this study (mastectomy and wide local excision) may obscure differences in psychological outcomes, as prior research indicates a greater psychological burden associated with mastectomy—especially with younger patients. Future studies may benefit from stratifying patients based on surgical intervention [22].

The single site and the cohort number might preclude generalization; however, to the best of our knowledge, this is still the largest exploratory study of its kind prospectively investigating psychosocial factors in older women with breast cancer.

## 6 Conclusion

In summary, psychosocial wellbeing was high in our cohort and was not associated with surgical versus non‐surgical treatment decision. Evidence in this area is limited, and further studies evaluating the importance of psychosocial factors as part of CGA are warranted to understand how best to use these measures. Treatment decision should be made following a discussion with the patient around life lengthening versus. QoL factors as aligning care with patient priorities can decrease treatment burden and unwanted health care. Careful consideration of the patient's preferences, consultation with the multi‐disciplinary team and application of CGA where practicable should all be part of this process.

## Author Contributions

Conceptualization: K.‐L. Cheung, A. Jahan, R.M. Parks. Methodology: K.‐L. Cheung. Investigation: R. Zahit, R.M. Parks. Data curation: R. Zahit, R.M. Parks. Analysis: G. Hindler. Writing ‒ original draft: G. Hindler, I. Alabaster. Writing ‒ review and editing: G. Hindler, I. Alabaster, R. Zahit, A. Jahan, D. Giza, H.M. Holmes, H. Blake, K.‐L. Cheung, R.M. Parks Supervision: K.‐L. Cheung, A. Jahan, R.M. Parks, H.M. Holmes, H. Blake, D. Giza.

## Ethics Statement

Ethical approval was obtained for this study from the Nottingham Research Ethics Committee (REC) 1, a part of the UK National Research Ethics Service (REC reference number 09/HO403/12).

## Conflicts of Interest

The authors declare no conflicts of interest.

## Supporting information

Supporting Information S1

## Data Availability

The data that support the findings of this study are available from the corresponding author upon reasonable request.
