# Supplementary material for: The Association Between Psychosocial Factors and Decision Making Regarding Primary Treatment in Older Women With Early‐Stage Breast Cancer
Source: Psychooncology. 2025 Jul 23;34(7):e70240. doi: 10.1002/pon.70240 (PMC12286772; doi:10.1002/pon.70240)
Supplement: Supplementary file 1 — Supporting Information S1 [file PON-34-e70240-s001.docx]

**Appendices**

*Appendix 1: Elements of CGA explored in this present study*
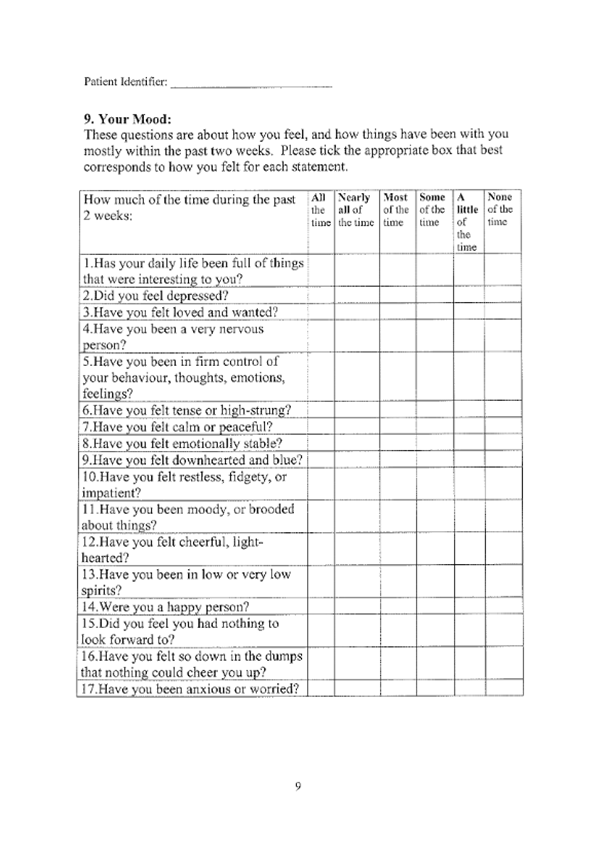


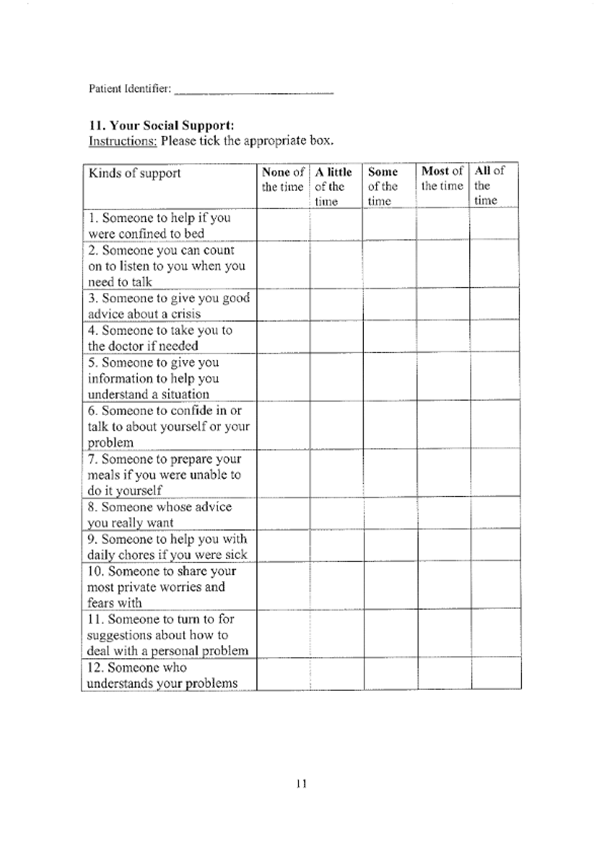


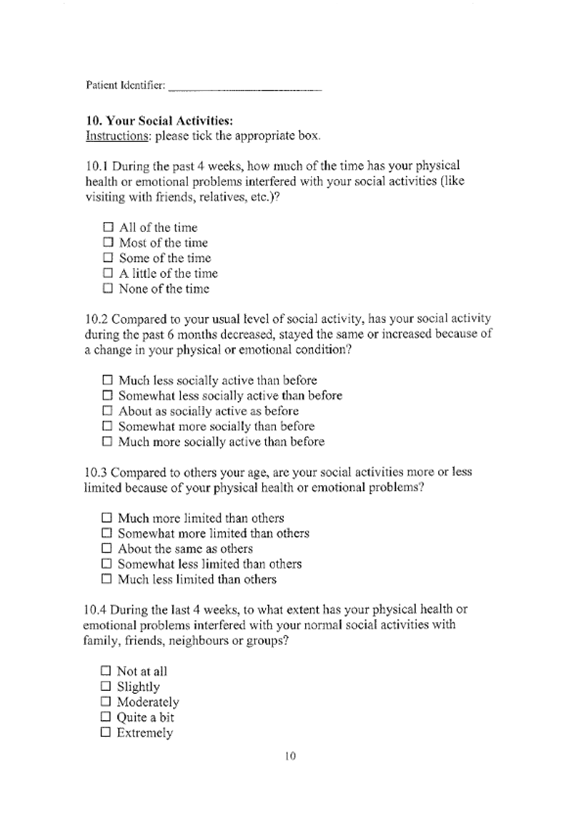


*Appendix 2: Individual Fisher’s exact test P values for components of the ‘Measures of psychosocial functioning’ section in the CGA.*

| **Question number** | **Question text** | ***p* value** |
| --- | --- | --- |
| 9.1 | Within the past two weeks has your daily life been full of things that were interesting to you? | 0.690 |
| 9.2 | Within the past two weeks did you feel depressed? | **0.019** |
| 9.3 | Within the past two weeks have you been a very nervous person? | 0.166 |
| 9.4 | Within the past two weeks have you felt loved and wanted? | 0.794 |
| 9.5 | Within the past two weeks have you been in firm control of your behaviour, thoughts, emotions, feelings? | 0.964 |
| 9.6 | Within the past two weeks have you felt tense or high-strung? | 0.890 |
| 9.7 | Within the past two weeks have you felt calm or peaceful? | 0.153 |
| 9.8 | Within the past two weeks have you felt emotionally stable? | 0.531 |
| 9.9 | Within the past two weeks have you felt downhearted and blue? | 0.333 |
| 9.10 | Within the past two weeks have you felt restless, fidgety or impatient? | 0.552 |
| 9.11 | Within the past two weeks have you been moody, or brooded about things? | 0.079 |
| 9.12 | Within the past two weeks have you felt cheerful, light-hearted? | 0.544 |
| 9.13 | Within the past two weeks have you been in low or very low spirits? | 0.847 |
| 9.14 | Within the past two weeks were you a happy person? | 0.326 |
| 9.15 | Within the past two weeks did you feel you had nothing to look forward to? | 0.117 |
| 9.16 | Within the past two weeks have you felt so down in the dumps that nothing could cheer you up? | 0.429 |
| 9.17 | Within the past two weeks have you been anxious or worried? | 0.478 |

*Appendix 3: Individual Fisher’s exact test p values for components of the ‘Measures of social activity’* *section of the CGA.*

| **Question number** | **Question text** | ***p* value** |
| --- | --- | --- |
| 10.1 | During the past 4 weeks, how much of the time has your physical health or emotional problems interfered with your social activities (like visiting with friends, relative, etc)? | 0.288 |
| 10.2 | Compared to your usual level of social activity, has your social activity during the past 6 months decreased, stayed the same or increased because of a change in your physical or emotional condition? | 0.582 |
| 10.3 | Compared to other your age, are your social activities more or less limited because of your physical health or emotional problems? | **0.005** |
| 10.4 | During the last 4 weeks, to what extend has your physical health or emotional problems interfered with your normal social activities with family, friends, neighbours or groups? | 0.762 |

*Appendix 4 - Individual Fisher’s exact test p values for components of the ‘Measures of social support’ section of the CGA.*

| **Question number** | **Question text** | ***p* value** |
| --- | --- | --- |
| 11.1 | Is there someone to help you if you were confined to bed? | 0.093 |
| 11.2 | Is there someone you can count on to listen to you when you need to talk? | 0.900 |
| 11.3 | Is there someone to give you good advice about a crisis? | 0.177 |
| 11.4 | Is there someone to take you to the doctor if needed? | 0.178 |
| 11.5 | Is there someone to give you information to help you understand a situation? | 0.256 |
| 11.6 | Is there someone to confide in or talk to about yourself or your problems? | 0.219 |
| 11.7 | Is there someone to prepare your meals if you were unable to do it yourself? | 0.809 |
| 11.8 | Is there someone whose advice you really want? | **0.012** |
| 11.9 | Is there someone to help you with daily chores if you were sick? | 0.881 |
| 11.10 | Is there someone to share your most private worries and fears with? | 0.743 |
| 11.11 | Is there someone to turn to for suggestions about how to deal with a personal problem? | 0.323 |
| 11.12 | Is there someone who understands your problems? | 0.595 |
